# Clinical characteristics and outcomes of the oldest old people with type 2 diabetes – perspective from a tertiary diabetes center in Thailand

**DOI:** 10.1186/s12902-016-0115-9

**Published:** 2016-06-01

**Authors:** Thewjitcharoen Yotsapon, Krittiyawong Sirinate, Wanothayaroj Ekgaluck, Vongterapak Somboon, Anuntakulnatee Tawee, Kittipoom Worawit, Nakasatien Soontaree, Himathongkam Thep

**Affiliations:** Diabetes and Thyroid Center, Theptarin Hospital, Bangkok, Thailand

**Keywords:** Oldest old, Type 2 diabetes, Thailand, Deintensification

## Abstract

**Background:**

Advance in medicine has led to an increase in life expectancy of elderly diabetic patients especially on the growing population called the “oldest old”, those in their mid-80s upwards. The aim of this study is to describe clinical characteristics and outcomes of “oldest old” patients in a specialized diabetes center.

**Methods:**

A retrospective review was conducted on medical records of type 2 diabetes who were older than 85 years at Theptarin hospital from September 2014 to August 2015.

**Results:**

During the study period, there were 143 oldest old diabetic patients who visited our hospital regularly. Of the 133 active follow-up patients (median time of follow-up 15 years, range 1–30 years), 70.7 % was female, the mean age of onset was 68.3 ± 11.5 years and duration of diabetes was 20.1 ± 11.1 years. According to the Charlson co-morbidity index (CCI), 35.3 % of patients were classified as having severe co-morbidities. The mean A1C, blood pressure, LDL were 6.7 ± 1.1 %, 132/65 mmHg and 80 ± 29 mg/dl respectively. 66.9 % of patients had tight glycemic control (A1C <7 %) while 12.0 % had poor control (A1C >8 %). Oral hypoglycemic agent (OHA) dual therapy was the most common treatment (26.3 %) followed by OHA monotherapy (22.6 %), insulin alone (19.5 %), diet therapy alone (12.7 %), and insulin plus OHA (8.3 %). Hypoglycemia was found in 10.5 % of patients in previous 12 months. Diabetic retinopathy, chronic kidney disease, cardiovascular disease, and stroke were presented in 23.4, 54.9, 15.8, 18.0 % of patients, respectively. Among patients whose received diabetic medications and resulted in very low level of A1C (A1C less than 6.0 %), only 20.0 % underwent deintensification.

**Conclusions:**

Our results revealed that real-world clinical outcomes of extreme elderly diabetic patients were diverse and being too “aggressive” diabetes treatment with older patients did occur frequently. Decision making in older people with diabetes is complex as chronic co-morbidities are very common.

## Background

Over decades, Thailand has developed to become the second largest economy in term of gross domestic product (GDP) in Southeast Asia, and 10^th^ in Asia [1]. At the same time, Thailand has become an aging nation. According to the United Nations projection [2], the proportion of people aged over 65 years in Thailand will increase from 10.5 % in 2015 to 26.6 % (17.6 million people) in 2030, and 32.1 % (20.5 million people) in 2040. In the mid-1980s, the term ‘oldest old’ referring to people aged 85 or older was introduced by gerontologists in an effort to focus attention on this growing segment of the population [3]. As the population continues to age since the term ‘oldest old’ was originally coined, it is important to note that the cut-off at age of 85 is fairly arbitrary and largely serves to create consensus and uniformity between studies. With the combination of aging population and diabetes epidemic, the number of elderly with type 2 diabetes (T2DM) will continue to rise.

Generally, the goal of diabetic treatment in the elderly is to maintain functional abilities and quality of life as well as to prevent diabetic complications. However, elderly patients have to cope not only with problems related to the management and treatment of diabetes, but also additional burden related to aging and associated co-morbidities [4]. Older adults with diabetes are at greater risk of other common geriatric syndromes such as depression, cognitive impairment, urinary incontinence, falls, polypharmacy, etc. [5–7]. These factors are responsible for deviation from guideline of therapy. Most clinical evidence on the management of diabetes was derived from randomized controlled trials but the evidence from these trials is often not applicable to older people as they are usually excluded [8]. The risk of hypoglycemia from treatments presents the greatest significant barrier to optimal glycemic control for the very old patients [9]. Furthermore, the physiology of aging changes their responses to therapy and other problems such as dementia, depression and frailty have a significant impact on the treatment and management of their diabetes [5].

The complexities of diabetes care associated with aging will continue to dominate clinical practice in the foreseeable future. Moreover, Decision making in older people with diabetes is complex and factors other than biomedical goals need to be addressed including co-morbidity and life expectancy. However, data for the oldest old group especially in diabetic patients are very limited. Therefore, the aim of this study is to describe the clinical characteristics and outcomes of oldest old with diabetes in a specialized diabetes center.

## Methods

We retrospectively reviewed medical records of all T2DM with age more than 85 years who were treated between September 2014 and August 2015 at Theptarin hospital, Bangkok, Thailand. Patients who were regularly follow-up at least 2 times during the study period (duration of 12 months) were included. Exclusion criteria included patients who were lost to follow-up in the previous 12 months. Demographic data, recorded glycated hemoglobin (A1C) in the previous 12 months, lipid profiles, serum creatinine, previous history of acute diabetic complications including hypoglycemia in the previous 12 months, chronic diabetic complications, and other co-morbidities during the study period were retrieved. In the absence of these data in the charts, telephone contact was attempted by diabetic nurse educators. Those who were diagnosed with diabetes when they were ≥ 65 years of age are referred to as elderly-onset diabetes and those diagnosed with diabetes at age < 65 years are referred to as long-standing diabetes group.

Retinopathy was detected with the regular dilated eye examinations by ophthalmologists annually. Nephropathy was defined as persistent proteinuria greater than 500 mg/24 h or microalbuminuria greater than 30 mg/24 h confirmed on at least 2 occasions, 3–6 months apart. Hypertension was defined as blood pressure >140/90 mmHg and hypercholesterolemia was defined as low density lipoprotein (LDL) cholesterol >100 mg/dL. For A1C level, we defined very low as less than 6.0 %, moderately low as 6.0 to 6.4 %, not low as 6.5 % or greater, and high as greater than 8.0 %. Safe margin of A1C was defined as all values that are neither low nor high (6.5–8.0 %) [10, 11]. Mild hypoglycemia was defined as hypoglycemia documented in the medical records which did not require additional support. Severe hypoglycemia was defined as hypoglycemia that required assistance to recover (e.g. ambulance call-out, hospitalization, supporter/relative assistance to aid recovery).

The complexity of co-morbidities was determined by the Charlson comorbidity index (CCI) which originally was developed to predict the one-year mortality based on comorbidity data [12]. This index score composed of 19 medical conditions and patients were divided into three groups: mild, with CCI scores of 1–2; moderate, with CCI scores of 3–4; and severe, with CCI scores ≥5. Diabetic medication deintensification was defined as discontinuation or dosage reduction of any diabetic medication during the last 6 months of study period after A1C level went below 6.5 %.

### Setting and study population

Theptarin Hospital is a private hospital in the central area of Bangkok and is one of the largest diabetes centers in Bangkok with over 2000 registered diabetic patients. Most diabetic patients are treated at diabetes clinic which has 12 diabetologists and a diabetes care team that includes diabetes nurse educators, dietitians and foot care specialists. Minority of elderly diabetic patients have regular follow-up by their primary physicians or a geriatrician. This retrospective study is approved by the Ethics board committee of Theptarin Hospital (No.02/2015).

### Statistical analysis

Continuous variables were presented as mean (SD) and categorical variables were presented as proportions. Comparisons between new-onset diabetes in elderly group and long-standing diabetes group were done using an unpaired Student’s t-test in continuous data and using a Chi-square test in categorical data. P-value ≤ 0.05 was considered statistically significant. All statistical analyses were conducted using the Statistical Package for the Social Sciences (version 17.0; SPSS, Chicago, IL, USA).

## Results

**Fig. 1:**
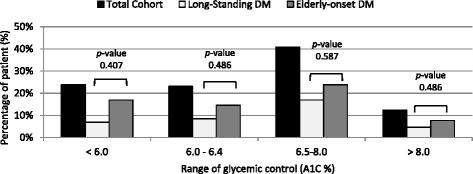
Range of glycemic control in oldest old diabetes patients

There are 143 oldest old diabetic patients visited our hospital regularly during the study period. The total number of these patients comprised of 5.0 % of the total 2859 registered patients in our hospital at the same period. Ten patients died from various medical illnesses during studied. Of the remaining 133 active patients (median time of follow-up 15 years, range 1–30 years), 70.7 % was female, the mean age of onset was 68.3 ± 11.5 years and duration of diabetes was 20.1 ± 11.1 years. Of these, 84 patients (63.2 %) were diagnosed with diabetes at age ≥ 65 years and formed the elderly-onset group (mean duration of diabetes 14.7 ± 8.3 years). The other 49 patients were diagnosed with diabetes at age < 65 years and formed the long-standing diabetes group (mean duration of diabetes 29.3 ± 9.1 years). Diabetic retinopathy, nephropathy, cardiovascular disease, and stroke were presented in 23.4, 54.9, 15.8, 18.0 %, respectively. Multi-morbidity was highly prevalent in these patients. On average, patients experience 4 concurrent chronic medical conditions. According to the Charlson co-morbidity index (CCI), 42.9 % of patients were classified as having moderate co-morbidities and 35.3 % of patients were classified as having severe co-morbidities. Cerebrovascular diseases were also prominent (18.0 %). Diagnosed dementia was found in 22.6 % of patients which almost half of them were bed-ridden. The demographic and general characteristics of the two patient groups of patients are summarized in Table 1. Metabolic controls and incidence of diabetic complications of both groups of oldest old diabetic patients were also showed in Table 1 and Fig. 1. Only the percentage of patients with diabetic retinopathy was statistically significantly higher in the long-standing diabetes group compared with the new-onset diabetes in elderly group (*P* = 0.014).

**Table 1:** Demographic data, pattern of diabetes treatment, and diabetic complications in oldest old diabetic patients

	Total patients (*N* =133)	Long-standing diabetes (*N* = 49)	Elderly-onset diabetes (*N* = 84)	p-value
Age (yrs)	87.7 ± 3.0	87.2 ± 3.0	88.1 ± 2.9	0.083
Female (%)	94 (70.7 %)	38 (77.6 %)	56 (66.7 %)	0.186
DM duration (yrs)	20.1 ± 11.1	29.3 ± 9.1	14.7 ± 8.3	<0.001
Age of onset (yrs)	68.3 ± 11.5	57.8 ± 9.3	74.3 ± 7.7	<0.001
Follow-up time (yrs)	15.1 ± 7.8	18.3 ± 8.3	13.2 ± 6.8	<0.001
BMI (kg/m^2^)	23.4 ± 4.2	23.4 ± 4.0	23.5 ± 4.4	0.971
Smoking status:				
-Never	116 (87.2 %)	45 (91.8 %)	71 (84.5 %)	
-Ex-smokers	15 (11.3 %)	4 (8.2 %)	11 (13.1 %)	
-Current	2 (1.5 %)	0 (0 %)	2 (2.4 %)	
Comorbidities				
-Myocardial Infarction	21 (15.8 %)	10 (20.4 %)	12 (14.2 %)	
-Stroke	24 (18.0 %)	10 (20.4 %)	14 (16.7 %)	
-Peripheral arterial disease^a^	26 (23.4 %)	9 (22.0 %)	17 (21.3 %)	
-Chronic kidney disease	73 (54.9 %)	25 (51.0 %)	48 (57.1 %)	
-Cancer	8 (6.0 %)	2 (4.1 %)	6 (7.1 %)	
-Dementia	30 (22.6 %)	12 (24.5 %)	18 (21.4 %)	
Charlson comorbidity index				
-Mild (1–2)	29 (21.8 %)	10 (20.4 %)	19 (22.6 %)	
-Moderate (3–4)	57 (42.9 %)	21 (42.9 %)	36 (42.9 %)	
-Severe (≥5)	47 (35.3 %)	18 (36.7 %)	29 (34.5 %)	
HbA_1c_ (%)	6.7 ± 1.1	6.8 ± 1.0	6.7 ± 1.1	0.454
Frequency of HbA_1c_ testings (times per year)	3.1 ± 1.4	3.2 ± 1.4	3.1 ± 1.4	0.714
Blood pressure (mmHg)	132 ± 16/65 ± 11	131 ± 16/64 ± 13	133 ± 15/67 ± 9	0.582
LDL (mg/dL)	80 ± 29	81 ± 34	79 ± 26	0.779
Diabetic retinopathy (%) ^b^	22 (23.4 %)	14 (37.8 %)	8 (14.0 %)	0.014
Pattern of diabetes treatment				
-Diet control alone	17 (12.8 %)	4 (8.2 %)	13 (15.5 %)	0.028
-Oral hypoglycemic agents	79 (59.4 %)	23 (46.9 %)	56 (66.5 %)	0.031
-Insulin therapy	37 (27.8 %)	22 (44.9 %)	15 (18.0 %)	0.013

^a^Data were available in 111 patients

^b^Data were available in 94 patients

During the study period, 125 patients (89 %) had A1C levels measured on average of 3 times (range 1–5 times) per year. The mean of A1C during the past 12 months was 6.7 ± 1.1 %. 66.9 percents of patients had tight glycemic control (A1C <7 %) while only 12.0 % of patients had poorly controlled diabetes (HbA1c >8 %). Very low level of A1C (less than 6.0 %), moderately low level of A1C (6.0 to 6.4 %), safe margin of A1C (6.5 to 8.0 %) were found in 23.3, 22.6, 39.8 %, respectively. The last measurement of mean blood pressure and LDL were 132/65 mmHg and 80 ± 29 mg/dl, respectively. Eighty-two percent of patients were on anti-hypertensive drugs and 76.7 % of patients were taking statins at the time of study. Regarding diabetic treatments, oral hypoglycemic agent (OHA) dual therapy was the most common treatment (26.3 %) followed by OHA monotherapy (22.6 %), insulin alone (19.5 %), diet therapy alone (12.7 %), and insulin plus OHA (8.3 %). For OHA-treated patients, metformin was the most commonly prescribed diabetic medication (38.3 %), followed by sulphonylurea (24.0 %), and DPP4 inhibitor (DPP4i) (24.0 %). Hypoglycemia was found in 10.5 % of patients in previous 12 months.

**Fig. 2:**
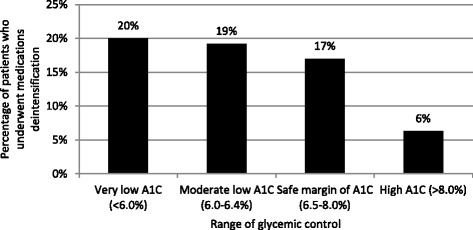
Rate of Medications Deintensification according to range of glycemic control

**Fig. 3:**
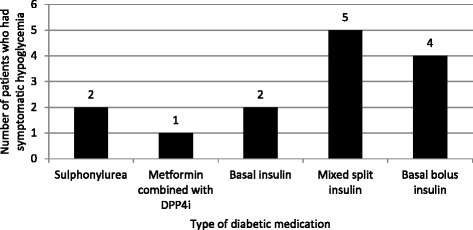
Incidence of symptomatic hypoglycemia in oldest old diabetes patients according to type of treatments

Among patients whose received diabetic medications and resulted in very low level of A1C (A1C less than 6.0 %), only 20.0 % underwent deintensification. The details of diabetic medications deintensification according to range of glycemic control were showed in Fig. 2. In the past 12 months, there were 2 patients who experienced severe hypoglycemia and 12 patients had symptomatic hypoglycemia (total rate of symptomatic hypoglycemia = 10.5 %). The occurrence of hypoglycemia was highest in the insulin-treated patients as shown in Fig. 3.

## Discussions

The world population is getting older in both developed and developing countries but this trend is occurring at a much faster rate in developing countries. A population is considered ‘aging’ when 10 % of the total population is 60 or older, and 7 % of the population is 65 or older. A society will turn into an ‘aged society’ and ‘superaged society’ once the proportion aged 65 or over is greater than 14 and 20 % of the population, respectively [2]. Thailand now stands in the forefront of high middle-income countries will move from being an ‘aging’ to an ‘aged’ society in less than a decade from now, and to a super-aged status in about half a decade. Among ASEAN countries, Thailand ranks second after Singapore in term of highest proportion of elderly people. This demographic change will produce wide-ranging effects on the country’s economy and social structure.

Frailty increases significantly with age. The ‘oldest old’ group (people aged 85 and older) therefore are usually contains the frailest members of society, who require support from health and social services to maintain good quality of life [13]. However, each individual varies widely in terms of demography, social and health characteristics [14]. Aging is unequal and is not a matter of chronological age alone. Also, there is a pronounced sex imbalance with women greatly outnumber men especially among the oldest old. With advancing age, older patients suffer more from various medical conditions including diabetes. Unfortunately, there is little evidence to guide recommendations for diabetes management in old age when issues of multi-morbidity, polypharmacy, frailty and dementia co-exist [10]. While older people tend to have normal hepatic glucose output, aging is associated with declining pancreatic islet cell function and lower insulin levels [5]. Elderly patients with diabetes mellitus can be divided into two subgroups: elderly age-at-onset (or senile diabetes) and younger age-at-onset (or long lasting diabetes) groups. Those who develop type 2 diabetes in old age are more likely to have near normal fasting glucose levels but significant postprandial hyperglycemia [15, 16]. In contrast to the new-onset diabetes in elderly, elderly with long-standing diabetes tend to develop more diabetic complications and require particular attention. As a result, the differences of demographic and clinical characteristics of these two groups lead to complexity in generalization of treatment or adherence to guideline. Moreover, most recommendations and guidelines tend to focus on a single disease rather than taking into account of managing chronic illness of aging populations. A recent global survey among leading worldwide diabetologists, the results from an example case of the oldest old revealed that “risk of hypoglycemia from treatment”, “life expectancy”, and “important comorbidities” were ranked among the top three parameters when set personalized target A1C [17]. The median target A1C had been proposed at 8.0 % (range 6.5–9.0 %) for the oldest old example case.

In the present study, there were heterogeneities of health status of oldest old diabetic patients and varieties of diabetes care patterns among diabetologists. About 13 % of oldest old diabetic patients especially in senile diabetes group can possibly be effectively managed with diet only. Even though almost 80 % of elderly-onset diabetic patients in our cohort had moderate to severe comorbidity conditions, their glycemic control were relatively better when compared with long-standing diabetic patients. The possible reasons might be explained by the benign nature of new-onset diabetes in elderly [5] and also various comorbidity conditions especially chronic kidney diseases in these patients might make patients more aware of eating habits.

Approximately one fourth of patients especially in long lasting diabetes group were on insulin treatment (mostly insulin analog). Although many insulin-based regimens have been used safely in elderly patients, hypoglycemia is a concern and presents a challenge for obtaining optimal glycemic control [18]. The main reasons for our diabetologists to use insulin in extreme elderly are comorbid conditions of patients which precluded from use of oral medications and failure of oral medications in bringing glycemic control to target. In our study, we found that symptomatic hypoglycemia tend to occur with insulin-treated patients. Mixed split insulin regimen was associated with the highest incidence of hypoglycemia. This finding might come from variable eating patterns of some elderly patients. In those patients, a basal-plus or basal-bolus regimen might be better options in patients who failed basal insulin regimen. As age, frailty, insulin treatment, and chronic kidney disease are known risk factors for hypoglycemia and hypoglycemic symptoms may be mistaken for other conditions associated more commonly with advanced ages, strategies to prevent hypoglycemia in very old patients should be emphasized. Focus should be on educating caregivers to recognize the signs and symptoms of hypoglycemia and to frequently perform home blood glucose monitoring.

For OHA-treated patients, metformin was commonly prescribed diabetic medication. This finding is contrast with a recent study from China which showed sulphonylurea was the most commonly used oral drug for elderly Chinese patients [19]. Although some concerns about an increase in the risk of metformin-associated lactic acidosis in very old patients and lack of data in this extreme aging population, the risk of lactic acidosis is very rare and preventable [20, 21]. In view of favorable profiles of metformin, it would continue to be used as a first line treatment in oldest old diabetes patients with adjusted dosage to renal function. In patients who are using sulfonylurea drugs, the presence and frequency of hypoglycemia should be evaluated at each visit. In our study, we also found that DPP-4 inhibitors were prescribed as the second most common drugs for the oldest old patients. A recent retrospective study on DDP-4 inhibitors prescribed to elderly diabetes patients (median age 70 years, range 60–92 years) found that median A1C improved from 8.3 to 7.4 % and DDP-4 inhibitors treated patients had a lower rate of documented hypoglycemia than the non-DPP-4 inhibitors treated group (3 vs. 8 %) [22]. Therefore, DPP-4 inhibitors would be an attractive choice for oldest old patients for its lower rate of hypoglycemia and neutral weight effects. Another class of adjuvant OHA which was popularly used in East Asian countries but not in our patients is alpha-glucosidase inhibitors (α-GI). Even though α-GI is an another attractive choice in elderly patients for low risk of hypoglycemia, gastrointestinal side effects and its clinical response which depends on preserved β-cell function limit its widespread use [23]. However, a recent population-based nationwide cohort study from Taiwan showed that α-GI (Acarbose) use reduced the risk of colorectal cancer in a dose-dependent manner [24]. The potential mechanism of this finding might be explained by the effects of Acarbose in reducing colonic transit time and changes in the fecal concentration of bile acids [25]. This interesting finding would deserve more study in a prospective study in elderly patients.

Older adults with diabetes are also at high risk for polypharmacy, functional disabilities, and common geriatric syndromes that include cognitive impairment, depression, urinary incontinence, falls, and persistent pain. Diabetes and its comorbidities are associated with Alzheimer’s disease, vascular dementia, and mixed dementia which the prevalence of dementia ranges from 18-38 % among those aged 85 and older, and from 28-44 % among those aged 90 and older [26, 27]. The increasing prevalence of mixed pathologies of dementia and susceptibility to side effects of treated medications among the oldest old make a difficulty in treatment of dementia in this age group. In our cohort, we found that more than one fifth of patients suffered from dementia and half of these patients had severe dementia with almost bed-ridden status. In theory, targets in frail older people with diabetes should focus on short-term day to day blood glucose levels to avoid hypoglycemia, rather than basing diabetes care around a long term A1C strategy. However, in a real-world setting, a large proportion of these patients are still receiving aggressive diabetic medications from specialists. This finding is similar to a recent study in US Veterans Health Administration which reported that only 27 % of patients who already had very low A1C or blood pressure underwent medication deintensification [28]. Therefore, overtreatment of diabetes in extreme age patients who had low life expectancy still prevails as showed in our study. Encouraging treating physicians to consider deintensification when appropriate should be focused as one of clinical performance index in diabetes care.

For chronic diabetic complications, our data also clearly showed that diabetic retinopathy (DR) was much more common in long-standing diabetes than elderly-onset diabetes (38 VS. 14 %). A recent study of DR prevalence in elderly Chinese population (mainly elderly-onset diabetes) revealed similar prevalence at around 16 % [29]. Therefore, even though the prevalence of DR is relatively low in elderly patients but long-standing diabetes patients suffer much higher rate due to long duration of diabetes. Timely screening and early treatment are still the basis of preventing blindness from diabetic retinopathy. Another medical condition which revealed a very high rate in oldest old patients was chronic kidney disease (CKD). The clinical and pathologic heterogeneity of nephropathy in type 2 diabetes especially in elderly patients reflect various causes of chronic kidney disease in this age group apart from metabolic consequence of diabetes. Optimal care for these patients is complex and best managed using comprehensive multi-factorial risk-reduction strategies with multidisciplinary team-based care [30-31]. Diabetes care should be individualized based on frailty, life expectancy, and co-morbid conditions.

The strengths of this study are that patients were followed up by the same diabetologists for a long period of time enabling us to collect details data and that this cohort study is the first comprehensive report on oldest old diabetes patients in Thailand. However, limitations do exist. First, the retrospective nature of the study resulted in relatively incomplete and missing data on some aspects such as neuropathy evaluation. Second, we used medical records to represent drug prescriptions rather than the actual dispensing data and also there was no data on patient adherence to treatment. Third, our study came from a single hospital in a private setting located in the central of Bangkok which limits the generalizability of the findings.

## Conclusions

In conclusion, managing the increasing population of extreme elderly people with diabetes represents a significant challenge. Our study revealed that real-world clinical outcomes of these diabetic patients were diverse and being too “aggressive” on diabetes treatment in older patients occurred frequently. Decision making in older people with diabetes is complex as chronic co-morbidities are very common in this group of patients. It is a challenge for policy makers in dealing with growing oldest old with diabetes population. Results from this retrospective study suggest opportunities to look into reducing overtreatment and rethinking about quality versus quality of life. This study also brings out the importance of recognizing heterogeneity of the population when it comes to treatment decision.

## Abbreviations

α-GI: alpha-glucosidase inhibitors; CCI: Charlson comorbidity index; CKD: chronic kidney disease; DR: diabetic retinopathy; DPP4i: DPP4 inhibitor; A1C: glycated hemoglobin; LDL: low density lipoprotein; OHA: oral hypoglycemic agent; T2DM: type 2 diabetes.
